# Patentdimensionen und die Entwicklung und Herstellung von Corona-Impfstoffen

**DOI:** 10.1007/s10273-021-2923-4

**Published:** 2021-05-17

**Authors:** Tobias Volpert, Marcel Riepe

**Affiliations:** grid.461668.b 0000 0004 0499 5893 VWL, Hochschule Hamm-Lippstadt, Marker Allee 76-78, 59063 Hamm, Deutschland

**Keywords:** O31, O38, K11

## Abstract

Hoffnungsträger zur Überwindung der Corona-Pandemie sind die potentiellen Impfstoffe. Um die Pandemie zu beenden, wäre ein weltweiter Einsatz dieser Impfstoffe nötig. Der knappe Impfstoff steht aber nur den reichsten Ländern der Welt zur Verfügung. Bei Innovationen wie den Corona-Impfstoffen ist es der Politik jedoch möglich, Patente auf unterschiedliche Art und Weise auszugestalten. Dabei macht es einen Unterschied, welche Dimension eines Patentsystems verändert wird, wenn die Politik das Ziel verfolgt, Fortschritt mit Hilfe von Patenten nicht nur zu fördern, sondern auch weltweit zugänglich zu machen.

## Abstract (en)

The process in which COVID-19 vaccines are developed is sequential and continuously accumulating. On the one hand, comprehensive patent protection is a necessary means to promote research and development. It yields additional returns that pay for the occasionally substantial amounts in R&D expenses. On the other hand, patents can be harmful in a sequential development process as they monopolise knowledge that might become a basis for further research. Economic policy cannot resolve this dilemma, but the various dimensions of a patent system can provide a starting point in mitigating a trade-off.

Anfang November 2020 befanden sich etwa 202 potentielle Corona-Impfstoffe in klinischen (47) oder präklinischen (155) Studien (WHO, 2020). Trotz der raschen Entwicklung erster Vakzine bleibt der Kampf gegen die Pandemie zäh. Seit dem Start der Massenimpfungen Ende 2020 sprechen Politiker:innen vom Licht am Ende des Tunnels. Allerdings handelt es sich um ein Licht, das derzeit nur in den reichen Ländern der Erde leuchtet. Zwar betont Entwicklungsminister Gerd Müller, dass der Impfstoff ein globales Gut sei, doch hatten sich Anfang Februar 2021 die reichsten 14 % der Weltbevölkerung mehr als die Hälfte der Impfdosen gesichert (WP, 2021). Ende Februar verfügten 130 Länder der Welt über keinen Impfstoff (Tagesspiegel, 2021). Die Pandemie trifft die gesamte Welt – doch trifft sie die Ärmsten schlimmer, zumindest aber länger als die Menschen in den Industriestaaten. Dabei sind sich Expert:innen einig, dass der Kampf gegen eine globale Pandemie nur gewonnen werden kann, wenn sie auch global bekämpft wird und die Menschen in allen Ländern zügig geimpft werden. Doch sind die zurzeit produzierten Impfstoffe kein globales Gut. Ausschlaggebend dafür ist, dass die Rezepte und Verfahren zur Produktion der Impfstoffe patentiert sind. Durch ein Patent wird ein Impfstoff zu einem rein privaten Gut.

Durch die Patentierung entsteht ein weitreichender Schutz für die Patentinhabenden. Während der Bestandsdauer des Patents existiert nicht nur ein zeitlich bzw. räumlich begrenztes Monopol für den bestimmten Impfstoff, sondern auch ein exklusives Herstellungs- und Verkaufsrecht für die entsprechenden Pharmaunternehmen (Stoianoff, 2020). Seine Eigentümer:innen verkaufen es meistbietend, womit der Großteil der Impfdosen in den entwickelten Ländern landet.

Am 4. März 2021 verkündeten die Regierungschefs der Länder Israel, Österreich und Dänemark den Aufbau einer Impf-Allianz. Die Motivation für diesen Schritt liegt zwar nicht in der Benachteiligung armer Länder beim Zugang zu Corona-Impfstoffen. Indirekt übten die Staatsoberhäupter aber Kritik an der Privatisierung dieses Wissens durch Patente. Denn das wesentliche Ziel der drei Länder ist Zusammenarbeit bei Forschung und Produktion von Corona-Impfstoffen, um Unabhängigkeit von den Pharmakonzernen zu erreichen (Sterkl und Backfisch, 2021).

Die möglichen Folgen solcher Patentierungen werden kontrovers diskutiert. Einerseits bieten Patente über ihren Schutz vor Nachahmung einen Anreiz für Investitionen in Forschung und Entwicklung neuer Impfstoffe (Stoianoff, 2020). Dies spielt angesichts der teilweise im dreistelligen Millionenbereich befindlichen Entwicklungskosten für medizinische Wirkstoffe eine signifikante Rolle (Schilling, 2014). Andererseits kommt es durch Patentierungen zu Rechtsstreitigkeiten und Konflikten, die dazu führen könnten, dass am Ende des Entwicklungsprozesses weniger

**Prof. Dr. Tobias Volpert** lehrt Angewandte Volkswirtschaftslehre, insbesondere International Economics an der Hochschule Hamm-Lippstadt.

**Marcel Riepe** ist wissenschaftlicher Mitarbeiter an der Hochschule Hamm-Lippstadt.

oder teurere Impfstoffe entstehen könnten (Dingermann, 2020). Die Diskussion gipfelt aktuell in einer Debatte darüber, Patentschutz für Corona-Vakzine vorübergehend offiziell durch die WTO auszusetzen, um anderen Herstellern als den Patentinhabenden die Möglichkeit zu geben, diese Impfstoffe lizenzfrei zu produzieren (Tagesspiegel, 2021).

### Impfstoffe als Ergebnis von Innovationssequenzen

Viele Innovationen – nicht nur im Bereich der Impfstoffentwicklung – bauen direkt auf bestehenden Wissensgütern auf. Eine Invention kann dann einen ganz neuen Technologiebereich eröffnen. Forschungsleistungen werden sich kumulieren und führen allmählich zu nützlichen Anwendungen. Als Beispiel für den Auslöser eines sich in mehreren Stufen allmählich kumulierenden FuE-Prozesses kann die Entschlüsselung eines Gens dienen, also genau das, was die Unternehmen Moderna und Biontech/Pfizer für die Entwicklung ihrer mRNA-basierten Corona-Vakzine gemacht haben.

Ein einzelnes Gen ist in der Regel an einer Vielzahl von Abläufen im Organismus beteiligt. Ist ein Gen identifiziert, ergeben sich damit weitreichende Entwicklungsmöglichkeiten in mehrere Richtungen. Zum einen lassen sich unterschiedliche Anwendungen, etwa ein Medikament, ein Diagnoseinstrument oder eine gezielte Gentherapie daraus ableiten. Zum anderen ist danach zu fragen, ob z. B. das die Krankheit Diabetes verursachende Gen gleichzeitig eine Rolle bei der Entstehung von Schwerhörigkeit spielt. Wo Zusammenhänge bestehen und wann sich einzelne Gene gegenseitig beeinflussen, ist vielfach noch unbekannt. Die Biowissenschaften stehen hier am Anfang eines sich in Zukunft nach und nach kumulierenden Forschungsprozesses. „Although a basic invention may have trivial value by itself, it may also be a technological breakthrough in that it generates great spillovers in the form of improvements likely to be far more valuable than the basic invention itself“ (Chang, 1995, 48).

Diese Spillover-Effekte entstehen auf unterschiedliche Weise: Komponenten einer patentierten Technologie können in eine neue importiert werden. Das bedeutet, dass eine vorhandene Technologie für eine andere, nicht konkurrierende Anwendung verwendet wird. Z. B. wurden mit der Lasertechnologie Verfahren für chirurgische Eingriffe ebenso wie zum Kartieren von Oberflächenstrukturen entwickelt. Daneben kann eine Basistechnologie weiterentwickelt werden. Eine lange Kette von Weiterentwicklungen illustriert z. B. die Geschichte der Prozessortechnologie von der x86-Architektur der 1980er Jahre hin zum Intel Xeon der Gegenwart. In beiden Fällen entsteht auf der Grundlage einer bestehenden Basistechnologie etwas Neues, mit bisher nicht dagewesenen Eigenschaften. Importation und Weiterentwicklung unterscheiden sich damit klar von einer Imitation. Durch Imitation werden Produkte geschaffen, die sich von bestehenden gar nicht oder in nicht wesentlichen Eigenschaften unterscheiden. Sie stellen ein vollkommenes oder nahes Substitut zur Ursprungserfindung dar.

Moderne Impfstoffe werden meist mit gentechnischen Verfahren hergestellt – auch diejenigen, die nicht mRNA-basiert sind (wie z. B. von AstraZeneca). Die Entwicklung von Impfstoffen gehört in jedem Fall in den Bereich der sich allmählich kumulierenden Forschung bzw. in den Bereich der Forschungssequenzen. Dies hat zwei Gründe:

1. Über Jahrzehnte wurde die Pharmaindustrie von großen multinationalen Unternehmen dominiert. Diese waren vollintegriert, d. h. sie umfassten von der Forschung bis zum Vertrieb alle Teile der pharmazeutischen Produktentwicklung. Mit der Etablierung der neuen, molekularen Biotechnologie änderte sich dieses Bild. Die Kommerzialisierung der Biotechnologie erfolgt häufig in kleinen, forschungsorientierten Start-ups. Diese fungieren seitdem zunehmend als Spezialfirmen, die sich auf spezielle Abschnitte in der Innovationskette der proteinbasierten Arzneiforschung konzentrieren. Ein aktuelles Beispiel ist die Firma Biontech aus Mainz. Sie entwickelte ein eigenes Verfahren, um Boten-Erbsubstanz in Körperzellen einzuschleusen. Diese Technik befindet sich erst seit wenigen Jahren in der Erprobung. Das ursprüngliche Ziel von Biontech war die Bereitstellung einer Krebs-Therapie, nicht die Produktion vom Impfstoffen gegen Coronaviren. Mittlerweile liefert Biontech erfolgreich eines der wichtigsten Corona-Vakzine.

2. Da Impfstoffe häufig mit einem komplexen Netz aus Patenten geschützt würden, die unter anderem verschiedene Bestandteile des Impfstoffs oder Einzelheiten des Herstellungsprozesses schützten, befürchten manche in Bezug auf eine OECD-Studie, dass der Technologietransfer unter diesen Patenten in einem solchen Ausmaß leiden könnte, dass es zu Problemen in der Branche führen würde (Reis, 2006).

In der Literatur finden sich unterschiedliche Kriterien, nach denen aufbauende FuE-Leistungen klassifiziert und charakterisiert werden können (z. B. De Laat, 1997, 8 f.; Merges und Nelson, 1994, 7). Für die folgende Analyse ist eine weitere Differenzierung der Topografie von Forschungsprozessen allerdings nicht von Bedeutung. Vielmehr genügt eine Unterscheidung zwischen der Schaffung von (nahen) Substituten bzw. Imitation und von neuen Produkten durch Importation und Weiterentwicklung, die im Folgenden unter dem Begriff aufbauende FuE bzw. FuE-Sequenzen zusammengefasst werden. Eine entscheidende Rolle bei der optimalen Förderung der verschiedenen Innovationstypen spielen die einzelnen Patentdimensionen.

## Dimensionen eines Patentsystems

Ein Patentsystem dient dem Staat als ordnungspolitisches Instrument zur Förderung von FuE. Es besteht aus Patentgesetzen, Ausführungsvorschriften und Verwaltungsprozeduren. Um gezielt Einfluss auf das Innovationsverhalten zu nehmen, kann der Staat das Patentsystem verändern und seinen Bedürfnissen anpassen. Dazu existieren unterschiedliche Ansatzpunkte innerhalb des Patentsystems; es ist multidimensional (De Laat, 1997).

O'Donoghue (1998, 657) identifiziert drei ökonomisch bedeutende Dimensionen eines Patentsystems: Die Länge bzw. der Geltungszeitraum eines Patents L, die Weite der Ansprüche aus einem Patent W und das Neuigkeitserfordernis (Non Obviousness Requirement: NOR), d.h. die minimal notwendige Erfindungshöhe, die eine Innovation aufweisen muss, damit ein Patent erteilt werden kann. O'Donoghue weist darauf hin, dass in der Literatur keine durchgängig einheitliche Nomenklatur für diese Dimensionen verwendet wird, stellt aber fest, dass dies die wichtigsten, ökonomisch relevanten Dimensionen sind.

### Geltungszeitraum L

Eine der wichtigsten Dimensionen eines Patents ist der Zeitraum, innerhalb dessen das Schutzrecht in Anspruch genommen werden kann. In den meisten Staaten beträgt dieser Zeitraum 20 Jahre ab dem Tag der Patentanmeldung. Allerdings sagt diese maximal mögliche Lebensdauer eines Patents nichts über den real in Anspruch genommenen effektiven Schutzzeitraum aus. Ein Patent ist jährlich durch das Entrichten einer Gebühr zu erneuern, sonst verliert das Patent seine Gültigkeit. Tatsächlich kommt es in der Praxis häufig vor, dass Patentinhabende auf eine zeitliche Verlängerung ihrer Ansprüche verzichten. In Deutschland beträgt der effektiv in Anspruch genommene Schutzzeitraum durchschnittlich 13 Jahre (Meier, 1998, 25). Gerade in der pharmazeutischen Industrie werden Patente hingegen häufig bis zum letzten Tag ausgenutzt. Dies liegt daran, dass sich einzelne Wirkstoffe bzw. Medikamente auch nach 20 Jahren noch gut verkaufen und nicht durch bessere ersetzt worden sind. Viele Pharmaunternehmen versuchen daher, den patentierten Wirkstoff kurz vor Ablauf des Patents so zu modifizieren, dass ein neues Patent, mit abermals 20 Jahren Gültigkeit, beantragt werden kann (Schumann, 2003, 95 f.).

In der Literatur wird meist angenommen, dass ein steigender Geltungszeitraum den erwarteten Ertrag aus einem Patent erhöhen wird und damit zu mehr Forschungsanreizen führt. Allerdings wird sich dieser Anreizeffekt mit zunehmender Gültigkeitsdauer abschwächen, da Erträge, die erst in der Zukunft realisiert werden können, aus heutiger Sicht an Attraktivität verlieren. Zudem wird es mit zunehmendem Alter der Innovation wahrscheinlicher, dass eine Imitation entsteht, durch die das Patent an Bedeutung verliert. Allerdings muss der Vorteil steigender Forschungsanreize aus einem Patent mit längerer Gültigkeitsdauer immer abgewogen werden gegen den Nachteil einer länger andauernden Monopolisierung eines öffentlichen Gutes. Das Abwägen dieser zwei Effekte mit dem Ziel, die optimale Gültigkeitsdauer zu bestimmen, war das erste große Problem, mit dem sich die Patentliteratur auseinandergesetzt hat (z.B. Nordhaus, 1967; Scherer 1972; Kaufer, 1989).

### Geltungsbereich W

Es finden sich unterschiedliche Definitionen für den Geltungsbereich W eines Patents. Grundsätzlich kann festgehalten werden, dass der Geltungsbereich oder die Schutzweite W ein Maß für die Rechtsansprüche darstellt, die sich aus einem Patent ergeben. W bestimmt den Umfang, in dem ein Patent die Verwendung der geschützten Idee durch Wettbewerber verhindert oder, andersherum, den Wettbewerbsvorteil, den der Patentinhaber gegenüber der Konkurrenz erhält. Obwohl die Bedeutung von W nicht unbekannt war (z.B. McGee, 1966), beachtete die Literatur zur Ökonomie von Patenten lange lediglich die Gültigkeitsdauer als wohlfahrtsrelevante Größe. Zwei entscheidende Beiträge änderten dies, indem sie W formal operationalisierten und erste wichtige Zusammenhänge aufzeigten, bei denen allein eine Variation von W den gesamtwirtschaftlichen Effekt von Patenten veränderte (Gilbert und Shapiro, 1990; Klemperer, 1990). Hierauf aufbauend entstand in den 1990er Jahren eine Literaturgruppe, die bis heute nicht nur unterschiedliche Wirkungen von W, sondern auch das Zusammenwirken von W und der Gültigkeitsdauer L eines Patents untersucht und dabei das Ziel verfolgt, einer optimalen Gestaltung von Patenten näherzukommen (z.B. Dam, 1994; Denicolo, 1996; Gallini, 1992; Klemperer, 1990; Gilbert und Shapiro, 1990; Lerner, 1994; Takalo, 1998; Whelan, 2000).

Die Definition, W lege den Schutzbereich eines Patents fest, ist recht allgemein gehalten. Van Dijk (1996) und O'Donoghue (1998) gehen weiter, indem sie W näher spezifizieren. O'Donoghue (1998, 657 f.) unterscheidet zwischen den Größen Lagging Breath und Leading Breath, Van Dijk (1996, 151 f.) spricht von Patent Breath und Patent Height. Beide meinen zwar den Schutz, den ein Patent gewährt, unterscheiden jedoch zwischen dem Schutz vor Imitation (Lagging Breath bzw. Patent Breath) und dem Schutz vor der Verwendung der patentierten Idee für Weiterentwicklung (Leading Breath bzw. Patent Height).

Die Länge eines Patents kann in Gesetzen exakt beschrieben werden. Hingegen ergibt sich die Weite häufig erst als Erfahrungswert aus regelmäßigen Entscheidungen der Patentgerichte, denen die Interpretation dieser Größe obliegt (Merges und Nelson, 1990, 840). Dabei existieren nicht nur Inkonsistenzen zwischen einzelnen Patentrechtsstreitigkeiten, auch im Zeitverlauf sind Änderungen der Weite von Patenten durchaus erkennbar. Garantierten z. B. US-Gerichte in den 1980er Jahren verhältnismäßig weite Patente, ließ sich seit Beginn der 1990er Jahre eine deutliche Einschränkung des Schutzumfangs registrieren (Lerner, 1994, 330 f.; sowie Plimier, 1998, 149 ff.). Die Informationen, die das kodifizierte Recht und dessen Handhabung durch die Gerichte über die Variable Weite liefert, bleiben in der Praxis damit unvollkommen, sodass immer wieder im Einzelfall eine mögliche Verletzung eines Exklusivrechts beurteilt werden muss (Llobet, 1999, 2).

Ein prominentes Beispiel aus dem Pharmabereich, das die Abgrenzungsproblematik von W verdeutlicht, ist die Potenzpille Viagra. Marktführer im Bereich der Potenzmittel war über viele Jahre der US-Konzern Pfizer, der diese Pille entwickelte. Pfizers Konkurrenten Eli Lilly und Bayer versuchten zu Beginn der 2000er Jahre, mit ähnlichen Produkten Marktanteile zu erobern. Die entscheidende Grundlage sowohl für Viagra als auch für die Konkurrenzprodukte ist bis heute die Wirkstoffklasse der sogenannten PDE-5-Hemmer, auf die Pfizer ein Patent hielt. Pfizer vertrat die Ansicht, dass die Ansprüche aus seinem Patent, also die Weite W für das PDE-5-Hemmer-Patent, so weit geht, dass Bayer und Eli Lilly die Produktion ähnlicher, aber nicht identischer Medikamente untersagt werden kann.

Das Beispiel zeigt, dass ein Patent umso wertvoller wird, je größer sein Schutzbereich W ist. Mit W steigt der monopolistische Vorteil des Patentinhabers, unabhängig davon, ob es sich im Detail um die Größe Imitation oder Weiterentwicklung handelt. Mit einem größeren W kann der Patentinhaber daher höhere Erträge aus dem Patent bzw. der geschützten Erfindung erwarten. Ob höhere Patenterträge bzw. ein größeres W zu mehr Forschungsanstrengungen führen, bleibt allerdings unklar. Steht eine Erfinderin oder ein Erfinder vor der Entscheidung zu forschen oder nicht zu forschen, so wird er sich bei einem steigenden zu erwartenden Patentertrag eher für die Alternative forschen entscheiden. Im Beispiel wird ein großes W Forschungsanstrengungen von Bayer und Eli Lilly allerdings verhindern. Dies wäre dann wenig sinnvoll, wenn im PDE-5-Hemmer-Patent Entwicklungspotenzial steckt. Für Pfizer als Patentinhaber ist nicht zu beurteilen, ob ein großes W und damit mehr Patenterträge zu mehr FuE führen oder ob ein kleineres W und damit entstehende Konkurrenz durch Bayer und Eli Lilly mehr Forschungsleistungen erzwingt. Eine eindeutige Aussage darüber, ob ein größeres W FuE-Leistungen generell erhöht, kann daher nicht getroffen werden (Volpert und Riepe, 2020, 777).

## Patentvoraussetzungen

Das US-Patentgesetz fordert, dass eine Erfindung neu (*novel*) und nicht offensichtlich (*non obvious*) sein muss, um sie für patentwürdig erklären zu können. Das Europäische Patentübereinkommen fordert 1. die Möglichkeit der gewerblichen Anwendung, 2. Neuheit und 3. eine erfinderische Tätigkeit, um eine Erfindung für patentfähig zu erklären. „Note that the United States notions of novelty and non-obviousness are roughly similar to those of newness and inventive step for Europe, but that the European industrial applicability condition does not appear in the United States Code: patentability conditions are more stringent in Europe“ (De Laat, 1997, 5).

Die ökonomische Patentliteratur fasst diese Erfordernisse meist in einer Größe zusammen, da nicht nur ein einzelnes, sondern alle in den Gesetzen benannten Erfordernisse simultan erfüllt sein müssen. Wird dieser Sachverhalt untersucht, finden sich Begriffe wie Patentfähigkeit oder NOR. Sie legen fest, ob eine neue Erfindung genügend neue Eigenschaften aufweist, um die Erteilung eines eigenen Patents zu rechtfertigen. Eine Erfindung muss demnach ein gewisses Maß an technischem Fortschritt liefern bzw. den Stand der Technik um ein Mindestmaß voranbringen, um als patentwürdig eingestuft zu werden. Dies wäre nicht der Fall, wenn die Erfindung offensichtlich, trivial oder in Expertenkreisen längst bekannt ist. Die in den Gesetzen benannten Erfordernisse seien im Folgenden unter dem Begriff Patentfähigkeit zusammengefasst, welche die minimal notwendige Erfindungshöhe, die zur Beanspruchung und Rechtfertigung eines Patents vorliegen muss, beschreibt.

## Schutzbereich eines Patents

Der gesamte Schutz, den ein Patent seinem Inhaber gewährt, wird damit auf drei verschiedenen Ebenen bestimmt: *Erstens* ist, im Rahmen der Wahl von W, festzulegen, wie weit der Schutz eines Patents geht bzw. ob und wann eine vermeintlich neue Erfindung als Imitation anzusehen ist. D. h., es ist eine maximale Zahl von Produkteigenschaften zu bestimmen, die imitiert werden dürfen, ohne dass es zu einer Verletzung des bestehenden Patents kommt. Hierfür sei der Begriff Breath verwendet. Je größer Breath ist, umso größer ist der Schutzbereich des Patents und umso geringer ist die Zahl von Produkteigenschaften, auf die der Imitator zugreifen kann. *Zweitens* ist, ebenfalls im Rahmen der Wahl von W, festzulegen, ob und wann eine vermeintlich neue Erfindung als Weiterentwicklung zu werten ist und damit, trotz der Verwendung einzelner Komponenten der bereits patentierten Erfindung, das bestehende Patent nicht verletzt. Hier sei der Begriff Height verwendet. Height bestimmt die minimale Zahl von neuen Produkteigenschaften, die eine Erfindung aufweisen muss, um nicht als Imitation des bereits bestehenden Patents gewertet zu werden. Drittens ist, im Rahmen der Wahl des NOR, zu klären, ob eine neue Erfindung genügend neue Eigenschaften aufweist, um die Erteilung eines eigenen Patents zu rechtfertigen. Der Begriff Patentfähigkeit scheint passend. Auch bei der Patentfähigkeit ist eine minimale Zahl von neuen Produkteigenschaften zu bestimmen, die eine Erfindung aufweisen muss, nun allerdings um als patentwürdig zu gelten.

Es stellt sich die Frage, ob Height und Patentfähigkeit den gleichen Sachverhalt prüfen. In beiden Fällen ist eine minimale Zahl von neuen Produkteigenschaften zu bestimmen. Eine Unterscheidung zwischen den beiden Dimensionen könnte daher überflüssig sein. Allerdings legt die Patentfähigkeit fest, wie die neue Erfindung gestaltet sein muss, um ein eigenes Patent zu rechtfertigen. Breath bezieht sich hingegen auf das bestehende Patent und legt fest, ob eine neue Erfindung dieses verletzt, indem es die erste Erfindung oder Komponenten der ersten Erfindung benutzt oder darauf aufbaut. Fraglich ist, ob diese Unterscheidung sinnvoll ist. Während Van Dijk (1996, 152) die Frage verneint, wird sie von O'Donoghue (1998, 658) bejaht.

Sollte immer dann, wenn mit einer neuen Erfindung die Breath eines bestehenden Patents verletzt wird, kein neues Patent möglich sein, dann wäre eine Unterscheidung zwischen den Dimensionen Breath und Patentfähigkeit tatsächlich überflüssig. Die Zahl minimaler neuer Produkteigenschaften müsste nur einmal überprüft werden. Tatsächlich kann es in der Realität aber vorkommen, dass eine Erfindung zwar patentfähig ist, die Zahl minimaler neuer Produkteigenschaften also aufweist. Gleichzeitig greift sie jedoch auf so viele Produkteigenschaften des bestehenden Patents zurück, dass sie die Breath des bestehenden Patents verletzt. Ein Patent wird in diesem Fall zwar erteilt, der Patentinhaber muss allerdings zur Verwertung seines Rechts zuvor eine Lizenz vom Inhaber des ersten Patents erwerben. Die Zwangslizenzen spielen in diesem Fall häufig eine wichtige Rolle. Sowohl Breath als auch die Patentfähigkeit legen also ein Minimum an neuen Eigenschaften fest, die eine neue Erfindung aufweisen muss. Breath bestimmt dann, ob ein bestehendes Patent verletzt wird, Patentfähigkeit bestimmt, ob ein neues Patent erteilt werden kann. Da beide Fälle unabhängig voneinander mit ja oder nein beantwortet werden können, ist eine Unterscheidung nützlich und sinnvoll.

Eine alternative Interpretation der Unterscheidung von Breath und Patentfähigkeit ist möglich: Die Breath legt fest, welche legalen Nutzungsmöglichkeiten einer patentierten Erfindung Konkurrenten haben. Sie bestimmt damit in erster Linie die Kosten für Importation, Imitation und Weiterentwicklung bereits patentierten Know-Hows. Denn auch wenn ein Patent den Gebrauch der Idee durch Konkurrenten verbietet, sind die Kosten für den Gebrauch nicht prohibitiv hoch, da prinzipiell immer die Möglichkeit besteht, Nutzungsrechte via Lizenzen zu erwerben. Die Patentfähigkeit wirkt hingegen auf der Ertragsseite einer Innovation. Indem die Patentfähigkeit festlegt, wann Rechtsschutz zulässig ist, bestimmt diese Größe, ab wann eine Innovation vermarktet werden kann.

Die Abgrenzung der einzelnen Patentdimensionen ergibt nur Sinn, wenn die Ordnungspolitik die Möglichkeit hat, die einzelnen Dimensionen gezielt zu steuern und zu verändern. O'Donoghue (1998, 657 f.) zeigt, dass dies tatsächlich der Fall ist und dass die einzelnen Patentdimensionen ihren Niederschlag im Recht und der die Gesetze interpretierenden Literatur finden: Die Dimension Patentfähigkeit leitet sich aus dem Erfordernis der Neuheit ab, die Dimension Breath aus dem Erfordernis der Offenbarung und die Dimension Height aus dem Erfordernis der gewerblichen Anwendbarkeit.

## Optimale Anreize zur Impfstoffentwicklung

Es stellt sich die Frage, ob die Wirtschaftspolitik bei der Bestimmung der einzelnen Patentdimensionen darauf zu achten hat, ob es sich bei der zu patentierenden Innovation um eine Einzelerfindung oder um eine Stufe innerhalb einer FuE-Sequenz handelt. Dabei ist zu beachten, dass ein Patent je nach der Weite seiner Gültigkeit in einem bestimmten Umfang und je nach seiner Gültigkeitsdauer für einen bestimmten Zeitraum die Verwendung der patentierten Idee als Input für Weiterentwicklungen durch Wettbewerber behindern wird. Das bestehende Patent wirkt damit nicht innovationsfördernd, sondern verbietet weiteren Fortschritt. Viele Modelle beachten diese zusätzliche Form sozialer Kosten nicht. Die Ergebnisse ihrer Analysen eignen sich daher nur für Einzelerfindungen und nicht für Innovationssequenzen. Der von ihnen geforderte optimale Patentschutz fällt damit für solche Innovationen mit Entwicklungspotenzial tendenziell zu groß aus (Denicolo, 1999, 845; Gilbert und Shapiro, 1990, 112).

Dieser Argumentation wird entgegengehalten, dass eine als Konsequenz nötige Abschwächung der exklusiven Rechte des Patentinhabers seine privaten Erträge und damit seine FuE-Leistungen und Erfindungen reduzieren wird. Basisinnovationen, die nicht gemacht werden, können aber auch keinen weiteren Fortschritt erzeugen (Eger et al., 1992, 93 ff.). Daher dürfen auch die später anfallenden zusätzlichen sozialen Kosten in Form ausbleibender, verminderter oder zeitlich verzögerter Folgeinnovationen nicht bei der Bestimmung optimaler Forschungsanreize berücksichtigt werden. Jede Stufe einer Innovationssequenz muss infolgedessen als Einzelerfindung betrachtet werden. Die Informationsfunktion von Patenten würde in diesem Fall bei der Festlegung der Patentdimensionen keine Rolle spielen.

Dieser Ansicht ist zweierlei zu erwidern. *Erstens* erscheint es realistisch, dass eine Ausdehnung der Gültigkeitsdauer eines Patents anders auf Folgeinnovationen wirkt, als eine Ausdehnung der Patentweite. Dann hängt zwar nicht die Höhe der Anreize für den Ursprungserfinder, wohl aber die Wahl der einzelnen Dimensionen des Patentsystems davon ab, ob es sich um eine Einzelerfindung oder eine Innovationssequenz handelt. Ein weites Patent mit kurzer Laufzeit wird zu weniger, ein dem Erfinder die gleiche Belohnung sicherndes enges Patent mit entsprechend langer Laufzeit wird zu mehr auf die Basisentdeckung aufbauendem Fortschritt führen. Wird technischer Fortschritt als generell wünschenswert angesehen, müsste eine Basisinnovation immer durch ein sehr eng definiertes Patent mit langer Laufzeit geschützt werden, sodass die Möglichkeit einer Verwendung des Grundlagenwissens für weiteren Fortschritt so wenig wie möglich blockiert wird.

*Zweitens* besteht auch für den Patentinhaber die Möglichkeit eines Ausbaus der Ursprungsidee, womit die Erfindung von ihm höher bewertet wird als in einer One-Shot-Betrachtung. Soll ihm oder ihr ein optimales Schutzrecht, das den minimal notwendigen Anreiz für FuE liefert, gewährt werden, muss dieser Wert potentieller Neuerungen aus dem patentierten Know-how berücksichtigt werden. Ist der temporäre Monopolgewinn aus dem Patent für die Grundlageninnovation größer als der minimal notwendige und besteht nicht die Gefahr, dass Wettbewerber eine Weiterentwicklung anbieten werden, sinkt für den Ursprungserfinder der Anreiz für selbst initiierte Verbesserungen. In diesem Fall wirkt nicht mehr, sondern weniger Schutz als Ansporn zum Ausbau der Basiserfindung (Denicolo, 1999, 845).

Matutes et al. (1996) bringen ein weiteres Argument. Gelingt einem forschenden Unternehmen eine fundamentale Entdeckung und erkennt dieses Unternehmen die sich daraus ergebenden Möglichkeiten, wird es versuchen, zunächst Anwendungen oder aufbauende Innovationen zu entwickeln, bevor die Erfindung zum Patent angemeldet und damit zwangsweise veröffentlicht wird. Das Unternehmen wird zunächst bemüht sein, gegenüber seinen Wettbewerbern einen Vorsprung in der Folgeentwicklung zu erreichen. Es entsteht eine „undesirable waiting period“ (Matutes et al., 1996, 60), da eine existierende nützliche Innovation nicht nutzenstiftend verwendet wird. Die Autoren zeigen, „that scope protection generates higher levels of welfare than does length protection because the period during which rivals can introduce applications of their own comes earlier and because the patentholder has more flexibility to decide when to exercise her property (patent) right.“ (Matutes et al., 1996, 61)

Business at OECD (BIAC, 2019) legt jedoch die Position dar, dass durch Patente sogar mehr Wettbewerb entstehen könnte, da Unternehmen durch Lizenzierung fremder Patente erst die Möglichkeit erhielten, in Produktion und Verkauf bestimmter Güter einzutreten, da ihnen sonst das Know-how oder bestimmte komplementäre Produktionsmethoden fehlen könnten (OECD und BIAC, 2019, 1). In Bezug auf eine mögliche Corona-Impfung könnten aus dieser Perspektive also kleinere Produzenten gegen die Zahlung von Lizenzgebühren ebenfalls einen bereits entwickelten Impfstoff produzieren, ohne selbst eine Forschungsabteilung einrichten zu müssen. Hieraus ergibt sich eine Make-or-Buy-Entscheidung in Bezug auf die Forschung, proprietäres Know-how bzw. weitere Vorarbeiten für bestimmte Unternehmen (Gassmann und Bader, 2006, 96 f.).

## Schluss

Im Ergebnis ist festzuhalten, dass es bei der Festlegung der einzelnen Patentdimensionen im Rahmen wirtschaftspolitischer Entscheidungen durchaus von Bedeutung ist, ob es sich um ein Patent für eine Einzelerfindung oder für eine Stufe innerhalb einer FuE-Sequenz handelt. Da sich FuE im Bereich der Impfstoffentwicklung und speziell in der molekularen Biotechnologie, mit deren Hilfe die neuartigen mRNA-basierten Corona-Vakzine entstehen, sequenziell entwickelt, ist zu hinterfragen, ob sich wirtschaftspolitische Entscheidungstragende und die ausführenden administrativen Stellen dieser Tatsache bewusst sind und ob sie dabei das Ziel verfolgen, die einzelnen Patentdimensionen so zu gestalten, dass Corona-Impfstoffe zum Wohle der Gesellschaft effizient entwickelt und zügig weltweit verfügbar gemacht werden. Im Vordergrund steht die Frage nach den einzelnen Patentdimensionen Breath, Heigh und NOR. Dabei dürfen politische Entscheidungstragende nicht alleine das Ziel verfolgen, die Erforschung wertvoller Impfstoffe zu fördern, die dann für lange Zeit alleine in den reicheren Ländern der Welt verkauft werden. Auch die optimale Diffusion patentierten Wissens muss berücksichtigt werden. Denn eines wird immer offensichtlicher: Die Menschheit wird die Corona-Pandemie nur in den Griff bekommen, wenn sie sie weltweit bekämpft.
